# The Role of *Hibiscus sabdariffa* L. (Roselle) in Maintenance of *Ex Vivo* Murine Bone Marrow-Derived Hematopoietic Stem Cells

**DOI:** 10.1155/2014/258192

**Published:** 2014-10-21

**Authors:** Zariyantey Abdul Hamid, Winnie Hii Lin Lin, Basma Jibril Abdalla, Ong Bee Yuen, Elda Surhaida Latif, Jamaludin Mohamed, Nor Fadilah Rajab, Chow Paik Wah, Muhd Khairul Akmal Wak Harto, Siti Balkis Budin

**Affiliations:** Program of Biomedical Science, School of Diagnostic and Applied Health Sciences, Faculty of Health Sciences, Universiti Kebangsaan Malaysia, Jalan Raja Abdul Muda Aziz, 50300 Kuala Lumpur, Malaysia

## Abstract

Hematopoietic stem cells- (HSCs-) based therapy requires *ex vivo* expansion of HSCs prior to therapeutic use. However, *ex vivo* culture was reported to promote excessive production of reactive oxygen species (ROS), exposing HSCs to oxidative damage. Efforts to overcome this limitation include the use of antioxidants. In this study, the role of *Hibiscus sabdariffa* L. (Roselle) in maintenance of cultured murine bone marrow-derived HSCs was investigated. Aqueous extract of Roselle was added at varying concentrations (0–1000 ng/mL) for 24 hours to the freshly isolated murine bone marrow cells (BMCs) cultures. Effects of Roselle on cell viability, reactive oxygen species (ROS) production, glutathione (GSH) level, superoxide dismutase (SOD) activity, and DNA damage were investigated. Roselle enhanced the survival (*P* < 0.05) of BMCs at 500 and 1000 ng/mL, increased survival of Sca-1^+^ cells (HSCs) at 500 ng/mL, and maintained HSCs phenotype as shown from nonremarkable changes of surface marker antigen (Sca-1) expression in all experimental groups. Roselle increased (*P* < 0.05) the GSH level and SOD activity but the level of reactive oxygen species (ROS) was unaffected. Moreover, Roselle showed significant cellular genoprotective potency against H_2_O_2_-induced DNA damage. Conclusively, Roselle shows novel property as potential supplement and genoprotectant against oxidative damage to cultured HSCs.

## 1. Introduction

Hematopoietic stem cells (HSCs) offer valuable source for cell-based therapy and regenerative medicine because of their self-renewing and multipotency capacity [1]. These features of HSCs are important for the maintenance of HSCs pool and subsequent lifelong haematopoiesis [2]. Prior to clinical usage,* ex vivo* expansion and maintenance of HSCs are crucial. However, the unique characteristics of HSCs are often altered once they leave the bone marrow niche, limiting the* ex vivo* expansion of HSCs. Moreover, another challenging factor affecting* ex vivo* expansion of HSCs is due to the inability to obtain optimal stimulation of HSCs proliferation while maintaining its undifferentiated state [3].

Previous studies have indicated that improper culture conditions [4], inappropriate microenvironment [5], and/or oxidative stress [6–8] could promote oxidative stress-induced genomic instability and apoptosis in cultured primary cells. Liu and colleagues [8] also reported that continued HSCs culture at normoxic condition exposed HSCs to oxidative stress and chromosomal instability. The survival and fate of hematopoietic progenitor cells in* ex vivo* system are reported to be dependent on multiple factors such as combination of cytokines cocktails, oxygen tension, and the presence of reactive oxygen intermediates/species (ROS/ROI). Oxidative stress and DNA damage are the most common factors that have been associated to cell death including bone marrow cells in which the events are believed to be mediated through excess production of reactive oxygen species (ROS) and reactive nitrogen species (RNS). Moreover, oxidative stress has been reported as one of the factors that promotes the premature senescence of* in vitro* cultured bone marrow-derived hematopoietic stem cells [9].

Various strategies have been developed to promote HSCs proliferation while maintaining their multipotency capacity. These include genetic manipulation [10], coculture with feeder cells [11], and addition of cytokines cocktails [12]. However, these approaches have their own drawbacks which include insertional mutagenesis associated with genetic manipulation [13], the xenogeneic contamination from animal-derived feeder cells [14], and the use of cytokines cocktails which are highly costly for long term cultures [12].

Efforts to overcome these limitations include the use of antioxidants [15–19]. A Japanese herbal medicine made from ten different herbs, TJ-48 (Juzen-taiho-to or Shi Quan-Da-Bu-Tang), showed positive effect on* ex vivo* hematopoietic restoration [20]. Also, proper dosages (0.1 *μ*M) of the antioxidant N-acetyl-cysteine (NAC) were found to decrease the rates of chromosomal change in HSCs [8], thus improving the survivability of HSC in* in vitro* cultures. Proliferation of human bone marrow cells has been successfully promoted with a combination of blueberry, green tea, catechin, carnosine, and vitamin D3 extracts [16]. Moreover, proliferative effect of epigallocatechin-3-gallate (EGCG) on* ex vivo* expansion of megakaryocytic progenitor cells before and after X-irradiation has been reported [15]. Interestingly, such claimed effects are believed to be mediated by the polyphenolic compounds, flavonoids that possess antioxidant activity. The usage of N-acetyl-cysteine (NAC) to diminish reoxygenation-associated DNA damage in bone marrow cells [9] and addition of recombinant purified catalase to mouse bone marrow that resulted in cell number increase [17] are the examples of antioxidant usage in bone marrow expansion. These findings emphasize potential utility of antioxidants to reduce oxidative stress, promote survival, and overcome chromosomal transformation on cultured HSCs, which serve important implications to the clinical usage. With these reported successes of antioxidants on the maintenance of HSCs, this study attempts to investigate for the first time the role of* Hibiscus sabdariffa* Linn. (also known locally as Roselle) on the cultured murine bone marrow-derived HSCs.

*Hibiscus sabdariffa* L. (Roselle) is a flavonoid-rich natural product and belongs to the family of Malvaceae, which is native to Asia (India to Malaysia) or Tropical Africa [21]. Roselle exhibits multiple biological activities in human health such as antioxidant [22], anticancer [23], antidiabetic [24], and hepatoprotective [25]. Most of the observed pharmacological effects of Roselle are believed to be mediated through its antioxidant properties. While many studies report possible beneficial activities of Roselle, little is known about its effects on hematopoiesis. Thus, this study aimed to determine the effect of Roselle on the* in vitro* maintenance of HSCs which include the analysis of cellular survivability, antioxidants, and oxidative stress status. Moreover, the potential genoprotective effects of Roselle on DNA damage using H_2_O_2_ as an induced model will be further evaluated.

## 2. Materials and Methods

### 2.1. Plant Material

Crude extract of* Hibiscus sabdariffa* L. was obtained by extracting calyces of red-leaf-type Roselle of UKMR-2 variety. Dried calyces of Roselle were obtained from Faculty of Science and Technology, Universiti Kebangsaan Malaysia (UKM). Briefly, maceration was initiated by cutting and blending dried calyces into a smooth paste, followed by a 1 : 2 dilution with distilled water. After boiling and filtration steps, the extract was freeze-dried with freeze dryer (FD5508 model, ilShin Lab, Korea) and finally stored in a dark and moisture-free container at 4°C until being tested for downstream analysis.

### 2.2. Experimental Protocols

All procedures involving the use of laboratory animals were reviewed and approved by the UKM Animal Ethics Committee (Ethics Approval Number: FSKB/BIOMED/2011/ZARIYANTEY/21-JULY/380-JULY-2011-JANUARY-2012-AR-CAT2). Bone marrow-derived hematopoietic stem cells were isolated from 30–35 g weighed male ICR mice by femur and tibia flushing. Briefly, the harvested cells were filtered through a 40 *μ*m cell strainer, centrifuged at 3000 rpm for 7 minutes, and resuspended in complete culture medium of Dulbecco's Modified Eagle Medium (DMEM) supplemented with 10% fetal bovine serum (FBS), 1% of Pen-Strep, 100 ng/mL stem cell factor (SCF), 10 ng/mL Interleukin-6 (IL-6), and 5 ng/mL Interleukin-3 (IL-3). Cell viability was determined by the trypan blue exclusion test and, routinely, around 1 × 10^7^ cells will be isolated from one mouse. Cell suspension was seeded into growth medium enriched with stem cells factor (SCF), Interleukin-6 (Il-6), and Interleukin-3 (Il-3) at density of 3.5 × 10^6^/mL for 24 hours prior to downstream application. After 24 hours of culture, cells were supplemented with Roselle extract at series of concentrations (31.25, 62.5, 125, 250, 500, and 1000 ng/mL of Roselle extract) for additional 24 hours. Cells without Roselle supplementation served as control group. After 24 hours of Roselle supplementation, cells were harvested for determination of cell viability using MTT assay. To our knowledge, no previous study concerning the effect of Roselle on* in vitro* culture of mouse bone marrow and hematopoietic stem cells has been reported. Thus, the selected concentrations of Roselle extract used in this study were based on Bickford et al. [16] who demonstrated for the first time that Blueberry and green tea extract ranging from 8 ng/mL to 500 ng/mL promoted proliferation of hematopoietic stem cells* in vitro* with greater stimulation observed starting from 125 ng/mL. Throughout the experiments, cells were maintained at 37°C in 5% carbon dioxide (CO_2_) incubator.

### 2.3. MTT Assay

Quantification of cell viability was achieved using the MTT assay. Firstly, 100 *μ*L of cells in complete growth medium was seeded into 96-well plates at seeding density of 1 × 10^6^ cells/mL. Then, cells were treated with Roselle extract at concentrations of 31.25, 62.5, 125, 250, 500, and 1000 ng/mL. After 24 hours of incubation, 20 *μ*L of 5 mg/mL MTT solution was added to the cells and incubated for another 4 hours at 37°C in a 5% CO_2_ incubator. Next, 150 *μ*L of dimethyl sulfoxide (DMSO) was added to the wells and further incubated for additional 15 minutes at 37°C and 5% CO_2_. Finally, the plates were measured at the absorbance of 570 nm and the defined OD correlates with the viability of the cells in culture.

### 2.4. Immunophenotyping for Stem Cell Antigen-1 (Sca-1) by Fluorescence-Activated Cell Sorting (FACS)

The analysis of surface marker expression for HSCs (Sca-1) was performed using BD FACS Calibur cytometer with Cell Quest Software (Becton, Dickinson and Company). Cells stained with the isotype matched antibody, namely, fluorescein isothiocyanate- (FITC-) conjugated anti-mouse IgG_2a_, were used as a control for the gating of positive cells. The analysis of Sca-1 expression was achieved using the fluorescein isothiocyanate- (FITC-) conjugated anti-mouse Sca-1 antibody. Both antibodies were from Miltenyi Biotec. Briefly, cell suspensions were stained with FITC-conjugated antibodies (~0.1 *μ*g/1 × 10^5^ cells) for 40 minutes at 4°C in the dark. Then, the cells were washed once by adding 1 mL of FACS staining buffer (PBS at pH 7.4–7.6, 2% heat inactivated bovine serum albumin, and 0.2% sodium azide) into each well and centrifuged at 1500 rpm for 5 minutes. The supernatants were decanted and cells were resuspended in 100 *μ*L of phosphate buffered saline (PBS). Finally, cells were analyzed using the flow cytometer and the true relative percentage of Sca-1 expression was obtained by deducting the negative control (IgG_2A_) value from the results of the tested samples. The absolute number of Sca-1^+^ HSC was calculated by multiplying relative percentage of Sca-1 expression with the determined total number of viable cell for each experimental group.

### 2.5. Determination of Antioxidant Status by Cellular Glutathione (GSH) Level and Superoxide Dismutase (SOD) Activity

The effect of Roselle extract on the antioxidant status of cultured bone marrow-derived hematopoietic stem cells was determined after 24 hours of supplementation at 125, 500, and 1000 ng/mL. Quantification of GSH which represents the intracellular content of nonenzymatic antioxidant was achieved using 5, 5′-dithiobis-2-nitrobenzoic acid (DTNB) according to the protocols as described by Ellman [26]. The reaction between GSH and DTNB formed a yellow-colored complex that was measured spectrophotometrically at 420 nm. GSH levels were expressed as nmol/mg. As for SOD, the activity was assayed according to the method as described by Beyer and Fridovich [27] based on the reaction of SOD with nitrotetrazolium blue chloride (NBT*·*2HCl). The NBT*·*2HCl reduction by SOD enzyme to NBT-diformazan was spectrophotometrically determined by reading the absorbance at 560 nm. One unit (1 U) of SOD was defined as the amount of SOD required for 50% inhibition of NBT*·*2HCl reduction.

### 2.6. Determination of Cellular Reactive Oxygen Species (ROS) Level

To study the effect of Roselle extract on the level of ROS, cells were exposed to Roselle at selected concentrations of 125, 500, and 1000 ng/mL. The level of ROS was measured with hydroethidine (HE) staining. Briefly, cells were harvested from each experimental group and centrifuged at 2500 rpm for 5 minutes at 15°C. Cells (1 × 10^6^/mL) were then loaded with HE (10 mM) for 15 min at 37°C with continuous shaking. ROS production was quantified by measuring the intensity of HE fluorescence using flow cytometry. ROS levels were then determined using mean fluorescence intensities and expressed as percentages of ROS producing cells.

### 2.7. Evaluation of DNA Damage by Alkaline Comet Assay

Genoprotective potential of Roselle extracts on cultured bone marrow-derived hematopoietic stem cells was studied. The level of DNA damage was assessed by using a standard protocol of alkaline comet assay with some modifications [28]. Briefly, cells were initially pretreated with Roselle extract at 500 ng/mL and 1000 ng/mL of Roselle for 24 hours. Then, cells were challenged with 100 *μ*M H_2_O_2_ for 10 minutes which was used as a model for oxidative-induced DNA damage. After treatment, cells were washed twice in Ca^2+^ and Mg^2+^-free PBS by centrifugation at 1200 rpm for 5 minutes at 4°C. Then, cells were mixed with 0.6% low melting point agarose and spread on a frosted microscopic slide precoated with 0.6% normal agarose. Slides were left cooled on ice for 5 minutes to solidify the agarose. Next, cells were lyzed by immersing the slides into lysis buffer (2.5 M NaCl, 100 mM Na_2_-EDTA, 10 mM Tris, and 1% Triton X-100) for 1 hour at 4°C. Slides were then equilibrated in electrophoresis buffer for 10 minutes, electrophoresed at 25 V for 20 minutes, and rinsed three times in neutralization buffer. Slides were stained with ethidium bromide (20 *μ*g/mL) and observed under the fluorescence microscope using 590 nm excitation filter. The percentage of tail DNAs and tail moment of 100 cells per slide were calculated using the CometScoreTM software.

### 2.8. Statistical Analysis

The results are reported as mean ± standard error mean (SEM) from three independent experiments. Results were analyzed using Student's *t*-test and one-way analysis of variance (ANOVA) with *P* < 0.05 being considered statistically significant.

## 3. Results

### 3.1. Effect of Roselle on the Survival of Cultured BMCs

**Figure 1:**
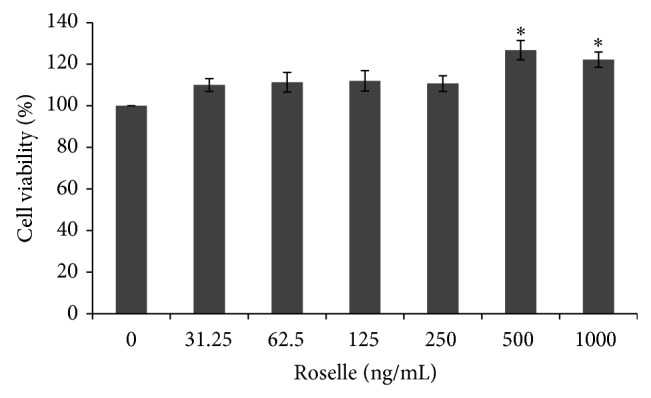
Effect of Roselle supplementation for 24 hours at various concentrations on the viability of cultured mouse bone marrow cells (BMCs). Results are means ± SEM of 3 replicates. ∗ indicates significant increase (*P* < 0.05) as compared to the control group.

The survival of cultured BMCs was increased in the presence of Roselle as compared to control group (Figure 1). Roselle supplementation enhanced the survivability of BMCs as evidenced from significantly higher (*P* < 0.05) viability of BMCs at 500 ng/mL (126.7 ± 4.6%) and 1000 ng/mL of Roselle (122.2 ± 3.7%). Addition of Roselle extract at lower concentrations than 500 ng/mL resulted in minimal promotion of BMCs survival as compared to control group.

### 3.2. Effect of Roselle on the Survival of Cultured Hematopoietic Stem Cells (Sca-1^+^)

**Figure 2:**
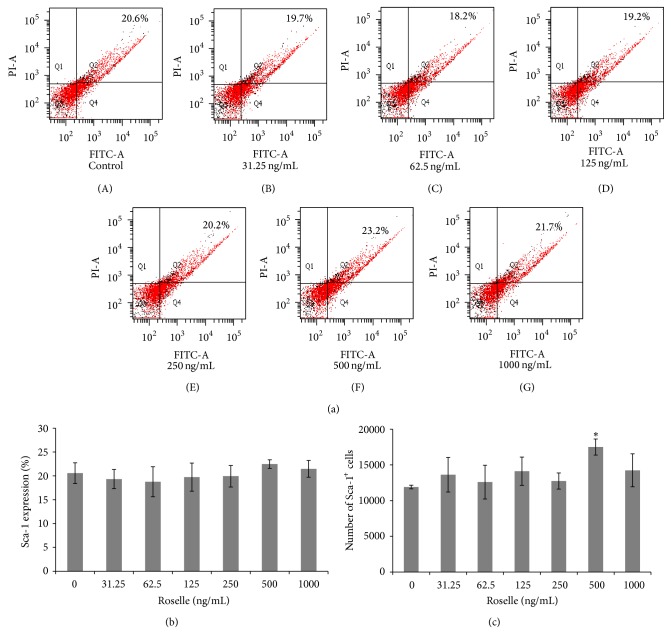
Effect of Roselle supplementation at various concentrations on the survival of Sca-1^+^ cells that represents subpopulation of mouse hematopoietic stem cells. (a) Representative flow cytograms of cells harvested from control (A) and 24 hours of Roselle supplemented groups (B–G). Cells were treated with anti-mouse FITC-Sca-1 monoclonal antibody and expression of surface antigen Sca-1 was analysed using a flow cytometer. (b) The percentage of Sca-1 expression from various experimental groups and (c) absolute counts of Sca-1^+^ cells from various experimental groups. The values are the mean ± SEM of 3 replicates. ∗ indicates significant increase (*P* < 0.05) as compared to the control group.

In order to investigate the effect of Roselle supplementation on the survival of cultured hematopoietic stem cells (HSCs), further phenotypic analysis on the expression of surface antigen marker selective for HSCs, namely, stem cell antigen-1 (Sca-1), was performed using a flow cytometer. As shown in Figures 2(a) and 2(b), the expression of Sca-1 marker was not affected after 24 hours of Roselle supplementation as demonstrated from nonremarkable changes on the percentage of Sca-1 expression in Roselle-added groups as compared to control group. The absolute counts of HSCs (Sca-1^+^) cells were calculated from the total number of cells harvested from the culture and the proportion of Sca-1^+^ detected in the harvested cells (Figure 2(c)). Overall, the addition of Roselle at 500 ng/mL resulted in the significant increase (*P* < 0.05) of Sca-1^+^ cells counts (1.75 ± 0.11 × 10^4^) compared to control group (1.2 ± 0.03 × 10^4^). Meanwhile, Roselle at concentrations of 31.25, 62.5, 125, 250, and 1000 ng/mL showed potential stimulatory effects on* ex vivo* Sca-1^+^ survivability as shown by higher numbers of Sca-1^+^ cells in Roselle supplemented groups than in control group.

### 3.3. Effect of Roselle on ROS Level, SOD Activity, and GSH Level in Cultured BMCs

**Figure 3:**
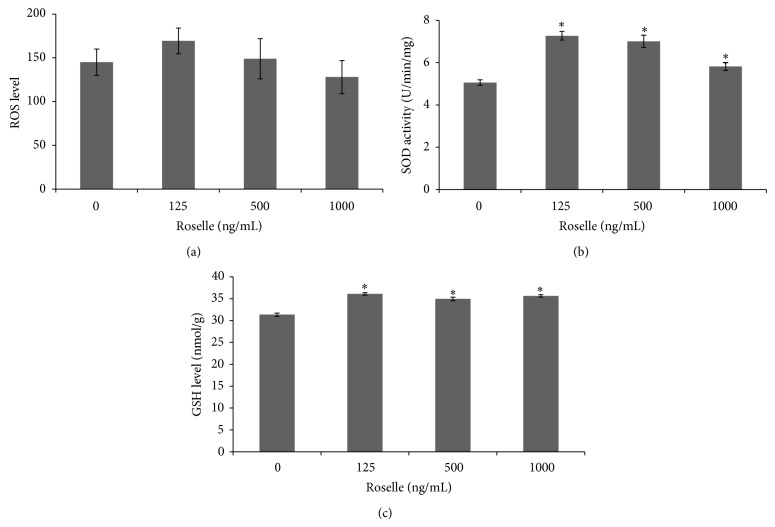
Effect of Roselle supplementation at various concentrations on the intracellular reactive oxygen species (ROS) production (a), superoxide dismutase (SOD) activity (b), and glutathione (GSH) level (c). Results are means ± SEM of 3 replicates. ∗ indicates significant increase (*P* < 0.05) as compared to the control group.

The role of Roselle in oxidative stress and antioxidant status in cultured BMCs was investigated by measuring the level of ROS and antioxidant capacity generated from cultured BMCs. As illustrated in Figure 3(a), there was no significant difference on the level of intracellular ROS between Roselle-added and control groups. However, the cells supplemented with 125 ng/mL of Roselle accumulated greater ROS as compared to other groups. The level of ROS for control group was 145 ± 14.9 while, for Roselle-added groups, the levels were 169.3 ± 14.7, 149 ± 23, and 128 ± 19 at Roselle concentrations of 125, 500, and 1000 ng/mL, respectively. As presented in Figure 3(b), Roselle supplementation significantly enhanced (*P* < 0.05) the activity of SOD in Roselle-added groups when compared to the control group. The SOD activity for control group was 5.05 ± 0.13 U/min/mg while, for groups that received 125, 500, and 1000 ng/mL of Roselle, the recorded activity was 7.267 ± 0.2 U/min/mg, 7 ± 0.29 U/min/mg, and 5.8 ± 0.18 U/min/mg, respectively. Results of GSH as illustrated in Figure 3(c) showed significant increase (*P* < 0.05) of the GSH levels in Roselle-added groups when compared to the control group (31.35 ± 0.38). In the presence of Roselle, the recorded levels were 36.1 ± 0.28 nmol/g, 34.9 ± 0.4 nmol/g, and 35.6 ± 0.28 nmol/g at Roselle concentration of 125, 500, and 1000 ng/mL, respectively.

### 3.4. Genoprotective Assessment of Roselle Against H_2_O_2_-Induced DNA Damage on Cultured BMCs

**Figure 4:**
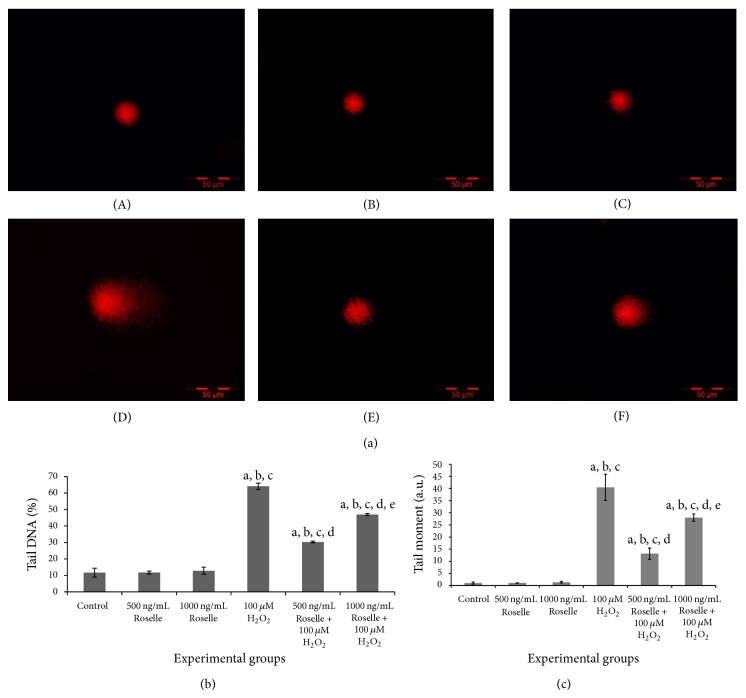
Effect of Roselle supplementation at various concentrations on H_2_O_2_-induced DNA damage of cultured BMCs. Pretreatment of cells with 500 and 1000 ng/mL of Roselle for 24 hours conferred significant protection against H_2_O_2_-induced DNA damage as measured through the percentage of DNA in tail (b) and tail moment (c) after 100 *μ*M H_2_O_2_ exposure for 10 minutes. Representative slides (a) of alkaline comet assay for various treatment groups are as follows: (A) control; (B) 500 ng/mL Roselle; (C) 1000 ng/mL Roselle; (D) 100 *μ*M H_2_O_2_; (E) 500 ng/mL + 100 *μ*M H_2_O_2_; (F) 1000 ng/mL + 100 *μ*M H_2_O_2_. Scale bar: 50 *μ*m. Results are means ± SEM of 3 replicates. Key: ^[a]^ *P* < 0.05, compared to control group; ^[b]^ *P* < 0.05, compared to 500 ng/mL Roselle; ^[c]^ *P* < 0.05, compared to 1000 ng/mL Roselle; ^[d]^ *P* < 0.05, compared to 100 *μ*M H_2_O_2_; ^[e]^ *P* < 0.05, compared to 500 ng/mL Roselle + 100 *μ*M H_2_O_2_.

Genoprotective potency of Roselle was further assessed using Comet Assay. As shown in Figure 4, pretreated cultured BMCs with 500 and 1000 ng/mL of Roselle for 24 hours were significantly protected against H_2_O_2_-induced DNA damage. This was evidenced through significantly lower (*P* < 0.05) percentage of DNA in tail (Figure 4(b)) and tail moment (Figure 4(c)) in Roselle-added groups as compared to 100 *μ*M H_2_O_2_-exposed group without Roselle pretreatment. Moreover, Roselle alone at 500 and 1000 ng/mL was not genotoxic as demonstrated from nonremarkable difference on the percentage of tail DNA and tail moment as compared to control group which is represented by cultured BMCs alone. Pretreatment with Roselle at 500 ng/mL showed the greatest protection capacity as evidenced from significantly lower (*P* < 0.05) percentage of tail DNA and tail moment in 500 ng/mL + 100 *μ*M H_2_O_2_ group as compared to 1000 ng/mL + 100 *μ*M H_2_O_2_ group. BMCs exposed to 100 *μ*M H_2_O_2_ resulted into 64.2 ± 2.0% of tail DNA while in the 24-hour pretreated BMCs with 500 and 1000 ng/mL of Roselle, significantly reduced (*P* < 0.05) percentage of tail DNA was observed with 30.3 ± 0.5% and 46.9 ± 0.6%, respectively. Meanwhile, the recorded tail moment for 100 *μ*M H_2_O_2_ group was 40.47 ± 5.4 which was significantly higher (*P* < 0.05) than in 500 ng/mL + 100 *μ*M H_2_O_2_ (13.15 ± 2.31) and 1000 ng/mL + 100 *μ*M H_2_O_2_ (28.0 ± 1.51) groups.

## 4. Discussion

Cultured hematopoietic stem cells offer invaluable source for therapeutic applications and functional studies. The proliferative potency and the ability to preserve the hematopoietic stem and progenitor's cells without genetic alteration during* ex vivo* culture are crucial requirements prior to its therapeutic application. Therefore, obtaining an* ex vivo* culture system that could support both requirements and subsequently minimizing risk of malignant transformation are fundamental to achieving proper maintenance of cultured HSCs. These efforts could ensure safety and efficacy of hematpoietic stem cells-based therapy.

Li and Marbán [29] demonstrated increased incidence of genomic abnormalities in cultured cardiac stem cells and embryonic stem cells that associated with increased ROS and high oxygen tension. However, addition of an optimal concentration of antioxidants and regulation of oxygen levels reduced ROS production and subsequent genomic abnormalities [8, 29]. This finding emphasizes the utility of antioxidants as a potential strategy to be employed to overcome limitations associated with cultured primary cells. In the present study, the effects of* Hibiscus sabdariffa* L (Roselle) on short-term murine BMCs and bone marrow-derived hematopoietic stem cells (Sca-1^+^) preservation, oxidative stress, antioxidant status, and DNA damage in* ex vivo* culture systems are described.

*Hibiscus sabdariffa* L. is an herbaceous shrub belonging to the family of Malvaceae [21, 30] and possesses various beneficial roles in human health as mediated through its antioxidant property [31–37]. This study has shown that short-term supplementation of Roselle for 24 hours can increase the survivability of murine bone marrow cells and the effect is remarkable at higher concentrations of Roselle. Moreover, Roselle supplementation preserves the expression of surface antigen marker for HSCs (Sca-1) and shows potential stimulatory effects on* ex vivo* Sca-1^+^ expansion with greater expansion at 500 ng/mL of Roselle. Moreover, greater survivability of the bone marrow cells than in Sca-1^+^ cells may reflect the effect of Roselle on the mature cell populations that are also present in the bone marrow cell cultures [16]. Of all the concentrations tested, 500 ng/mL of Roselle most consistently produced significantly increased survivability when examining proliferation of bone marrow cells and Sca-1^+^ cells. Thus, it may suggest that Roselle when supplied to culture medium at specific concentrations can act as a growth promoter to increase the proliferation of primary mouse bone marrow cells and hematopoietic stem cells (Sca-1^+^) without significant loss of phenotype. Overall, our finding may indicate potential usefulness of Roselle supplementation in optimizing* ex vivo* hematopoietic stem/progenitor cells expansion system that are usually maintained for 3-4 doubling times [38]. Thus, it is possible that longer supplementation of Roselle at optimal doses is required to obtain remarkable and lasting effects on hematopoietic stem/progenitor's preservation and proliferation* ex vivo*. Although the increase in the number of Sca-1^+^ could be due to either an increase in the rate of proliferation or inhibition of apoptosis, the precise mechanism is currently unclear. Therefore, future study should identify the precise mechanism that results in the observed events.

The survival and fate of hematopoietic stem/progenitor cells in* ex vivo* system are reported to be dependent on multiple factors. These include combination of cytokines cocktails, oxygen tension, and the presence of reactive oxygen intermediates/species (ROS/ROI) [39]. Numerous studies have demonstrated that excessive ROS may cause cellular damage such as oxidation of membrane lipids, DNA breakdown, and decomposition of proteins [18, 35, 40]. However, ROS are regarded as key regulator in intracellular signaling for HSC proliferation, differentiation, and mobilization [41, 42]. ROS, at physiological level, are vital to maintain genomic stability in stem cells [29]; at intermediate levels, they block self-renewal and stimulate stem cell differentiation [43]; at high levels, severe oxidative damage that is induced by ROS leads to programmed cell death or apoptosis [41].

Oxidative stress-induced apoptosis has been evidenced in several types of stem cells [33, 44]. The role of antioxidant in the inhibition of apoptosis in stem cells [19] and in the maintenance of HSC has been previously reported. As reviewed in Naka et al. [45], excessive level of intracellular ROS induces HSCs to undergo senescence or apoptosis which subsequently impair HSC self-renewal. However, the effects were overcome by the treatment with antioxidant which indicates that excess ROS levels are closely related to the HSC failure. Moreover, a study conducted by Jang and Sharkis [46] revealed a novel mouse HSC phenotype that displayed similar HSC cell-surface markers but possessed two distinct populations that are not identical based on their intracellular content of ROS, namely, ROS^low^ HSC and ROS^high^ HSC. The association of ROS contents and HSC function is further highlighted with ROS^low^ HSC that demonstrated greater long-term self-renewal ability than the ROS^high^ HSC. More interestingly, functional activity of ROS^high^ HSC was restored following the addition of an antioxidant, N-acetyl-L-cysteine (NAC). This finding emphasized the fundamental role of ROS and potential application of antioxidants in controlling the long-term self-renewal ability of HSC and functional hematopoiesis.

Whether Roselle could minimize oxidative stress in cultured bone marrow and hematopoietic stem/progenitor cells or not, the level of ROS and antioxidant capacity generated from the cultured cells in the presence or absence of Roselle were measured. The findings show that Roselle is able to remarkably increase both the GSH level and SOD activity in Roselle-added group, which reflects the antioxidant action of Roselle in primary culture of bone marrow and hematopoietic progenitor cells. However, Roselle does not significantly affect the production of ROS. Although it is reported that ROS and inducible nitric oxide synthase (iNOS) are high in CD34^+^ cells [47], it is possible that hematopoietic progenitors do not express iNOS/ROS at high level at this stage of 48-hour* ex vivo* culture. Thus, no comparable effect was observed within group despite of enhanced antioxidant level/activity in Roselle-added groups. Our finding may suggest that Roselle may be able to restore the intracellular antioxidant system to protect HSCs from ROS-induced oxidative damage.

Several studies showed that* ex vivo* culture system exposes hematopoietic stem/progenitor cells to greater ROS-mediated genetic damage. Thus continuous efforts to overcome DNA damage of cultured hematopoietic stem/progenitor cells are crucial to avoid potential unchecked mutation and genomic instability of the cells during* ex vivo* preservation [45]. In addition to DNA damage, excess ROS was shown to cause telomere dysfunction and subsequent premature replicative senescence on the cultured human fibroblasts [48]. Because the long-term self-renewal capacity of HSC is determined by the maintenance of chromosomal telomeres, it is therefore possible that excess intracellular ROS production during* ex vivo* maintenance of the cells not only will cause DNA damage but also will cause potential acceleration of telomeric shortening and replicative senescence of hematopoietic stem/progenitor cells.

Several lines of evidence have reported the role of antioxidants on inhibiting genetic alteration in hematopoietic cells. A significant level of aneuploidy in enriched population of murine HSCs (Lineage-Sca-1^+^ c-Kit^+^) following short-term* in vitro* culture has been reported and the finding may indicate potential genetic alteration in hematopoietic cells [8]. Addition of the antioxidant N-acetyl-cysteine (NAC) is however able to decrease the emergence of chromosomal change in cultured HSCs, suggesting potential role of antioxidants to reduce oxidative stress changes and chances of chromosomal transformation. In the present study, the genoprotective potency of Roselle on oxidative damage was studied using H_2_O_2_ as the ROS-induced model and the level of DNA damage was assessed using Comet Assay. After preincubation of murine bone marrow cells with Roselle for 24 hours, a significant protection against oxidative damage by H_2_O_2_ was observed with 500 ng/mL Roselle conferring better protection than the higher dose of 1000 ng/mL. Moreover, Roselle supplementation for 24 hours shows nonremarkable changes in the level of DNA damage compared to control group as determined by percentage of tail DNA and tail moment. This latter finding demonstrates potential cellular genoprotective effect of Roselle, which is important for further application in HSC maintenance in culture.

Maintenance of genomic stability in stem cells is dependent on the physiological levels of intracellular ROS [29, 45] and low oxygen levels (hypoxic) promote the survival and self-renewal of HSCs during* in vitro* culture [49, 50]. This finding indicates that karyotypic abnormalities in cultured hematopoietic cells are associated with oxidative stress. The association of oxidative stress and genomic stability of cultured HSCs was further evidenced when addition of NAC to the cells that were cultured under normoxic conditions was able to significantly reduce the occurrence of genomic instability as compared to nontreated group. More interestingly, the protective effect was only remarkable at low dosage of NAC as compared to higher dosage which may explain that a narrow range of ROS levels is required for maintaining optimal genomic stability of cultured HSCs.

## 5. Conclusions

In conclusion, this study demonstrates for the first time that the addition of Roselle during culture shows a cell-genoprotective potential and modulates proliferation and intracellular antioxidant system of mouse bone marrow and hematopoietic stem/progenitor cells (Sca-1^+^), without causing a remarkable loss of surface marker expression for HSCs (Sca-1). It was determined that, in short-term culture, addition of Roselle at specific concentration can increase the number of murine bone marrow cells and hematopoietic stem/progenitor cells which could be mediated through the increase of the antioxidant capacity. These results indicate that cultured murine bone marrow cells and hematopoietic stem/progenitor cells respond to exogenous agents on proliferation and that Roselle antioxidant flavonoids can restore their intracellular antioxidant system. Suppression of DNA damage and enhanced survival of hematopoietic progenitor/stem cells in culture can improve the quality of donor cells for transplantation. More detailed studies to explore the relationship between Roselle and HSCs are required so that the application of antioxidant supplementation to the primary culture can be realized more effectively. Moreover, further studies to determine optimal concentrations of Roselle that could support long-term maintenance of cultured HSC remains to be explored. Overall, this study would help to discover novel property of Roselle as potential supplement and genoprotectant against oxidative damage to cultured HSCs.
